# A single-centre analysis of the clinical characteristics of non-tuberculous mycobacterial lung disease caused by rare species

**DOI:** 10.5588/ijtldopen.26.0123

**Published:** 2026-06-15

**Authors:** K. Fujita, O. Kanai, N. Fujimoto, S. Yoshimura, S. Toyama, T. Ito, T. Imakita, I. Oi, Y. Yamamoto, K. Tanizawa

**Affiliations:** 1 Division of Respiratory Medicine, Centre for Respiratory Diseases, National Hospital Organization Kyoto Medical Centre, Kyoto, Japan;; 2 Department of Infectious Diseases, National Hospital Organization Kyoto Medical Centre, Kyoto, Japan;; 3 HiLung Inc., Kyoto, Japan.

**Keywords:** NTM, Japan, infectious disease, pulmonary disease

Dear Editor,

Non-tuberculous mycobacteria (NTM) comprise approximately 200 distinct species, of which around 30 have been documented to exhibit pathogenicity in humans.^1^ The epidemiological landscape of NTM demonstrates considerable geographical heterogeneity,^2,3^ but global surveillance data consistently indicate that the *Mycobacterium avium* complex (MAC) accounts for approximately 60% of all NTM isolates, followed by *M. abscessus* at 20%, *M. kansasii* at 10%, and other miscellaneous species collectively representing the remaining 10%.^3,4^ Rare NTM species, each with a detection rate below 1%, are encountered infrequently in routine clinical practice and present significant diagnostic and therapeutic challenges.^4^ Current clinical practice guidelines, including those established by the American Thoracic Society (ATS) and the European Respiratory Society (ERS), provide treatment recommendations exclusively for MAC, *M. abscessus*, and *M. kansasii*,^2,5^ but therapeutic guidance for other NTM species remains conspicuously absent from these evidence-based frameworks, reflecting the paucity of robust clinical trial data and limited understanding of the behaviour of these organisms.^6^ Consequently, the clinical management of NTM lung disease caused by rare species is largely left to the discretion of individual clinicians, both domestically and internationally.^4,7^ The rarity of these infections precludes large-scale randomised controlled trials, considerable heterogeneity in antimicrobial susceptibility patterns among rare species, and a lack of systematic documentation of treatment outcomes.^6,8^ This study aimed to provide valuable empirical evidence to inform clinical decision making in the absence of formal guidelines.^2,6^

This retrospective cohort study was conducted at the NHO Kyoto Medical Centre, Japan. For this investigation, rare NTM species were operationally defined as those with detection frequencies of approximately 1% or less. Patients were deemed eligible for inclusion if they fulfilled the diagnostic criteria of the 2007 ATS/IDSA statement and the 2020 ATS/ERS/ESCMID/IDSA clinical practice guidelines.^2,5^ We assessed the distribution of rare NTM species, the proportion of patients who met the diagnostic criteria caused by rare species, and the proportion of patients who underwent therapeutic intervention and clinical outcomes. The frequency and severity of treatment-related adverse events were categorised according to the Common Terminology Criteria for Adverse Events (CTCAE) version 5.0.^9^ This study was approved by the Institutional Review Board of the NHO Kyoto Medical Centre (approval number: 26-065).

Seventeen rare NTM species were detected in 45 patients (see Table 1). The five most frequently detected NTM species were *M. gordonae*, *M. lentiflavum*, *M. fortuitum*, *M. mageritense*, and *M. paragordonae*. Of the 45 patients, 17 (37.8%) met the diagnostic criteria. Diagnoses were based on sputum culture (n = 11) or bronchoalveolar lavage (n = 6). The median age was 76.5 years (range, 56–89 years), and six patients (35.3%) were female. The radiological patterns were nodular bronchiectatic (n = 13), fibrocavitary (n = 2), or unclassifiable (n = 2). The most common co-morbidities were malignancy (29.4%) and COPD (23.5%). Clinical course of the treated and untreated patients is shown in Table 2. Treatment was initiated in 5 cases, whereas the remaining 12 cases were managed with observation alone. The reasons for observation were patient refusal (n = 7), uncertain treatment regimens (n = 2), and severe clinical co-morbidities (n = 3). The treatment regimens used were azithromycin (AZM) and ethambutol (EB) for *M. gordonae* and *M. colombiense*; AZM + meropenem for *M. shimoidei*; clarithromycin (CAM) + levofloxacin for *M. fortuitum*; and CAM + EB + rifampicin for *M. gordonae*. Among the five treated patients, sputum smear conversion after treatment was achieved in three (60.0%) patients, with a median treatment duration of 11 months (range, 1–24 months). Of these three patients who achieved sputum smear conversion, two achieved sputum conversion within 2 months and one within 1 month. In the five treated patients, improvement was observed in two (40.0%), no change in one (20.0%), and deterioration in two (40.0%): diarrhoea was observed in three patients and was the most frequently reported adverse event, all Grade 2, followed by nausea, observed in two patients, all Grade 2. Next, a skin rash was observed in a patient, graded as Grade 1. Among the 12 untreated patients, 5 (41.7%) remained unchanged, whereas 7 (58.3%) deteriorated.

**Table 1.** Identified rare non-tuberculous mycobacterial (NTM) species.

NTM species	All patients (n = 45)	Patients meeting the diagnostic criteria^A^ (n = 17)	Clinical outcome for patients meeting the diagnostic criteria^A^ (n = 17)		
			Improvement	No change	Deterioration
*M. gordonae*	8 (17.8)	3 (17.6)		2 (11.8)	1 (5.9)
*M. lentiflavum*	8 (17.8)	4 (23.5)		1 (5.9)	3 (17.6)
*M. fortuitum*	7 (15.6)	2 (11.8)	1 (5.9)	1 (5.9)	
*M. mageritense*	4 (8.9)				
*M. paragordonae*	4 (8.9)	3 (17.6)		1 (5.9)	2 (11.8)
*M. mucogenicum*	2 (4.4)				
*M. paraense*	2 (4.4)				
*M. chelonae*	1 (2.2)				
*M. colombiense*	1 (2.2)	1 (5.9)	1 (5.9)		
*M. mantenii*	1 (2.2)	1 (5.9)			1 (5.9)
*M. paraffinicum*	1 (2.2)				
*M. septicum*	1 (2.2)				
*M. shimoidei*	1 (2.2)	1 (5.9)			1 (5.9)
*M. shinjukuense*	1 (2.2)	1 (5.9)			1 (5.9)
*M. szulgai*	1 (2.2)				
*M. wolinskyi*	1 (2.2)				
*M. xenopi*	1 (2.2)	1 (5.9)		1 (5.9)	

A ATS/ERS/ESCMID/IDSA diagnostic criteria for non-tuberculous mycobacteria.

**Table 2.** Clinical course of the treated and untreated patients.

Clinical course	Treated patients (n = 5)	Untreated patients (n = 12)
Outcome		
Improvement	2 (40.0)	0 (0.0)
No change	1 (20.0)	5 (41.7)
Deterioration	2 (40.0)	7 (58.3)
Treatment regimen		
AZM + EB	2 (40.0)	—
CAM + EB + RFP	1 (20.0)	—
CAM + LVFX	1 (20.0)	—
AZM + MEPM	1 (20.0)	—
Duration of treatment	11 (1–24)	—
Sputum smear conversion after treatment	3 (60.0)	—
Reason for no treatment		
Patient refusal	—	7 (58.3)
Uncertainty regarding the optimal treatment regimen	—	2 (16.7)
Presence of severe co-morbidities	—	3 (25.0)

AZM = azithromycin; EB = ethambutol; RFP = rifampicin; CAM = clarithromycin; LVFX = levofloxacin; MEPM = meropenem.

This study demonstrates that a highly diverse range of rare NTM species can be detected, even within a single centre. However, most detections did not fulfil the diagnostic criteria,^2^ supporting the interpretation that a substantial proportion of rare NTM isolates represent colonisation rather than clinically significant disease. Among the patients meeting the diagnostic criteria,^2^ only 29.4% received treatment. This low treatment proportion likely reflects the combination of i) limited evidence and lack of guideline-endorsed regimens for most rare species and ii) patient-related constraints in real-world practice. In our cohort, the median age was 76.5 years, and co-morbidities such as COPD (23.5%) and malignancy were common. These host factors increase vulnerability to adverse events, drug-drug interactions, and treatment discontinuation during prolonged multidrug therapy, and therefore may raise the threshold for treatment initiation even when diagnostic criteria are met. The predominance of patient refusal further underlines the importance of shared decision making for rare NTM lung disease, where the uncertainty in expected benefits is often substantial.

Radiologically, the NB type accounted for 76.5% of cases meeting the criteria, whereas the FC type was uncommon (11.8%). Although NB is frequently viewed as less aggressive than FC, our CT-based follow-up showed deterioration in 58.3% of untreated patients, indicating that the NB-predominant type due to rare species is not necessarily indolent. Conversely, the outcomes in treated patients were heterogeneous: CT improvement occurred in 40.0% and deterioration in 40.0% of patients, with sputum smear conversion achieved in 60.0% of patients. These mixed results likely reflect inter-species differences in antimicrobial susceptibility and virulence as well as the pragmatic and non-standardised nature of regimen selection. Importantly, the outcome assessment in this study relied on CT changes, whereas the standardised composite outcome definitions for NTM lung disease incorporated microbiological and clinical domains.^9^ Small-scale clinical reports concerning specific rare NTM species are occasionally encountered; however, the evidence for this has not yet been established.^10–12^ In the absence of established regimens for rare species, macrolide-based combinations analogous to MAC therapy were used in this cohort. This practice is understandable given that the current major guidelines focus primarily on MAC, *M. abscessus*, and *M. kansasii*, leaving clinicians to individualise management for other species.^2,5^ Nevertheless, extrapolation from MAC should be undertaken cautiously because rare species include both slowly and rapidly growing mycobacteria with markedly different drug susceptibility patterns and resistance mechanisms. Therefore, species-level identification, careful longitudinal assessment of the disease trajectory, and susceptibility-guided regimen construction, where available, are essential for optimising outcomes in rare NTM lung disease.

This study has several limitations. First, it was retrospective, had a small sample size, and was conducted at a single centre, which may reduce the generalisability and introduce selection bias in diagnostic testing and treatment decisions. Second, the treatment regimens were heterogeneous and selected pragmatically without a standardised protocol, making it difficult to attribute outcomes to specific drugs or combinations. Third, clinical outcomes were assessed primarily using CT findings. Microbiological endpoints, such as sustained culture conversion based on serial sputum cultures and symptom trajectories, were not uniformly available and may have led to outcome misclassification. Finally, the follow-up duration and timing of imaging assessments were not standardised.

In conclusion, a wide diversity of rare NTM species was detected at a single centre. However, most isolates did not fulfil the diagnostic criteria and were presumed to represent colonisation rather than active disease. Among the patients meeting the diagnostic criteria, fewer than one third received treatment, reflecting substantial uncertainty regarding optimal regimens and important patient-related constraints.
